# Attitudes and awareness of regional Pacific Island students towards e-learning

**DOI:** 10.1186/s41239-021-00248-z

**Published:** 2021-03-11

**Authors:** Joel B. Johnson, Pritika Reddy, Ronil Chand, Mani Naiker

**Affiliations:** 1 School of Health, Medical and Applied Sciences, CQUniversity, North Rockhampton, QLD Australia; 2 Department of Computing Science and Information Systems, Fiji National University, Suva, Fiji

**Keywords:** COVID-19, Distance education, First-year undergraduates, Pandemic, Preparedness

## Abstract

The rise of online modes of content delivery, termed e-learning, has increased student convenience and provided geographically remote students with more options for tertiary education. However, its efficacy relies upon student access to suitable technology and the internet, and the quality of the online course material. With the COVID-19 outbreak, education providers worldwide were forced to turn to e-learning to retain their student base and allow them to continue learning through the pandemic. However, in geographically remote, developing nations, many students may not have access to suitable technology or internet connections. Hence it is important to understand the potential of e-learning to maintain equitable access to education in such situations. This study found the majority (88%) of commencing students at the University of the South Pacific owned at least one ICT device and had access to the internet. Similarly, most students had adequate to strong ICT skills and a positive attitude toward e-learning. These attitudes among the student cohort, in conjunction with the previous experience of The University of the South Pacific in distance education, are likely to have contributed to its relatively successful transition from face-to-face to online learning as a result of the COVID-19 pandemic.

**Supplementary Information:**

The online version contains supplementary material available at 10.1186/s41239-021-00248-z.

## Introduction

The use of ICT (Information and Communication Technologies) to deliver educational content and learning support now forms a widespread and accepted norm of many institutes in the higher education sector across the world (Latchem, 2017; Sharma et al., 2020; Wu, 2016). Its growing leverage in developing countries is assisting in bringing the quality, sustainability, accessibility and delivery of education on par with those of the developing countries. The ICT based innovations and tools from the higher education institutes in the developing countries have shown promising and significantly positive results (Reddy et al., 2016, 2020c; Sharma & Reddy, 2015; Sharma et al., 2019a, 2020, 2018b).

Indeed, ICT is now an integral part of most institutes rather than supporting tools (Bhuasiri et al., 2012; Irfan et al., 2018). The use of e-learning is of particular importance for reaching students in geographically remote locations and improving the equity towards the delivery of education systems (Graham, 2019). However, certain barriers remain towards the use of ICT and e-learning as tools for enhancing educational equity (Lim et al., 2020; Yang et al., 2018). Firstly, some students in remote locations or those living in low socio-economic status (SES) situations may not have access to the appropriate technology or resources required to successfully participate and engage in the e-learning process (Yang et al., 2018). For instance, students may not be able to afford equipment such as laptops or desktop computers, or be able to afford internet access plans with sufficient bandwidth and data allowance for video streaming or other data-heavy applications (Chillemi et al., 2020). This is more prominent in developing countries such as those in the Pacific region (Reddy et al., 2017, 2019, 2020a, 2020b; Sharma et al., 2018a, 2018b). Hence in many of the Pacific Island Countries (PICs), government initiatives have been implemented to provide school students with laptops and tablets in order to allow them to access online resources and obtain assistance with their schoolwork (Reddy et al., 2016, 2017, 2020b, 2020c). Similarly, students may only have limited access options for accessing the internet (e.g. through a mobile phone network) (Huang et al., 2017), especially in more remote locations (Beinicke & Bipp, 2018; Milakovich & Wise, 2019), or may be restricted to accessing the internet in certain locations such as local remote campuses and/or education centres, such as in Samoa and Tonga (Reddy et al., 2017; Sharma et al., 2019a, 2020).

In addition, studies have reported a "digital divide" among students of different socio-economic classes, with primary and secondary students from a low SES background typically having poorer ICT literacy (Scherer & Siddiq, 2019), albeit to a lesser degree compared to other domains such as reading and mathematics. It is important to note that this divide is due to differences in their literacy (i.e. familiarity and capability) with ICT devices, not their access to ICT devices per se (Cotten et al., 2014). However, in many instances, their lower ICT literacy would likely result from restricted access to e-technology at home or in rural schools in developing countries. Previous surveys show that over 50% of the population in the South Pacific now has access to the internet as a result of improved network infrastructure and the reduced cost of ICT devices (Reddy et al., 2017). However, these studies also highlight the variation in device ownership amongst the student population as a result of the financial, social and cultural challenges that exist in the PICs (Raturi, 2018; Reddy et al., 2019; Sharma et al., 2019a, 2020, 2019b). Education institutes and governments across the PICs have attempted to assist the students facing such challenges through interventions such as the provision of one free laptop or tablet per child, and equipping schools with adequate internet connections and computing devices (Sharma et al., 2020, 2019b). Similarly, the SchoolNet Project in Samoa and Tonga was funded by the Asian Development Bank (ADB) and implemented by the government to enhance learning outcomes for secondary students and improve knowledge sharing through equitable ICT access (ADB, 2019). Nevertheless, despite such assistance from a number of stakeholders, digital literacy in the PICs remains a challenge. One recent study at a regional university in the South Pacific found 73% of the freshmen to have high to very high digital literacy competencies (Reddy et al., 2020d); whereas another study conducted on high school students in Fiji found 61% to have very low to average competency in digital media literacy. Students will only continue to use digital technology if they have a positive attitude and perception towards technology-enabled learning (Reddy et al., 2020a, 2020d). In turn, the students will only have a positive attitude towards technology-enabled learning if they possess the competencies needed to use the technology in question (Reddy et al., 2020a). Hence the advocacy of digital literacy in the PICs has just begun.

However, challenges remain worldwide with the use of e-learning. Students who participate in e-learning may not necessarily receive the same educational benefits as those who enrol in face-to-face classes. It is typically more difficult and time-consuming to ask the lecturer a question through an online forum than in a face-to-face lecture or tutorial situation (Heirdsfield et al., 2011). Conversely, it may be more difficult for the educator to explain more complex topics online, where it is more difficult to capture non-verbal aspects used in teaching, such as fine hand gestures or annotations on a whiteboard (Arasaratnam-Smith & Northcote 2017; Phirangee & Hewitt, 2016). Additionally, a lack of real-time feedback from the students makes it more difficult for the lecturer to tailor their lecture content to suit the class's overall learning style. In other words, most lecturers must significantly transform their teaching style from that which they use in face-to-face classes in order to deliver the content in an online format effectively (Kebritchi et al., 2017). Similarly, another issue for content providers is the increased difficulty in designing effective online activities and assessments (Kebritchi et al., 2017). This can result in lower student engagement and interaction, combined with an increased chance of online cheating (Olt, 2002). From the perspective of both lecturers and students, it is often more of a challenge to build good verbal and non-written communication skills in an online format (Lalande, 1995), which are typical graduate attributes sought after by prospective employers. Overall, the failure of educators to meet these challenges may result in a poorer learning experience for their online cohort of students, which in turn has implications for student achievement and student loyalty to their education provider (Pham et al., 2019).

Some subjects, such as introductory chemistry units, include teaching certain practical skills to students in order to prepare them for future, more advanced units (Chandra & Sharma, 2018; Naiker et al., 2013; Naiker & Wakeling, 2015; Wakeling et al., 2014). This is typically delivered as a compulsory "residential" or "practicum" block for online/distance students, typically ranging from several days to a week in length, rather than the short, weekly lab sessions typically used with on-campus students. However, in many subjects which do not contain a compulsory practical component, gaining hands-on practical experience may still be quite useful for students to solidify their theoretical knowledge and gain greater insight into the practical implications of what they have learnt. For example, this situation is valid for computer programming courses. Such benefits are not available to students studying solely online.

However, online learning is not without its benefits (Chandra & Sharma, 2018). In many instances, it provides the students with greatly increased flexibility, allowing them to learn wherever and whenever they want (Hollenbeck et al., 2005; Kilburn et al., 2014). There are considerable benefits associated with self-paced (Bhuasiri et al., 2012), self-directed and personalised learning. In addition to the increased equitable access to education afforded by online learning (Graham, 2019), students can replay lectures to solidify their knowledge of the content, and benefit from online peer feedback (van Popta et al., 2017) and peer mentoring (Fayram et al., 2018). Moreover, there are increasing volumes of high-quality resources, including OERs (open educational resources), available for students undertaking e-learning, even if their host institution does not necessarily provide these resources. Other ventures such as MOOCs (massive open online courses) and diagnostic tools, such as the Online Mathematics Diagnostic Tool (Sharma et al., 2019a, 2020) can also increase educational equity for students unable to attend prestigious universities (Gardner & Brooks, 2018; Littlejohn et al., 2016).

## Literature review

The innovations in information and communication technology (ICT) and its integration into the education sector has massively impacted the education process, particularly in higher education. New learning methods such as web-based or Internet-based delivery modes have evolved into a broad range of learning modes, including e-learning, m-learning (mobile learning), tablet-learning and flipped classrooms (Ansong-Gyimah, 2020; Reddy et al., 2020b). In the recent years, e-learning has become one of the most trending learning methods in academia (Bhuvaneswari & Dharanipriya, 2020). E-learning has been defined as the use of information and communication technologies (ICT) in education which continues to evolve to meet the needs and demands of the students (Bhuvaneswari & Dharanipriya, 2020), E-learning involves the use of technology and web platform to create a two-way platform for communication and discussion between students and teachers, where student-to-student discussions enhance social learning, and teachers provide a scaffolded learning experience for students via timely feedback (Layali & Al-Shlowiy, 2020), E-learning encompasses a broad set of applications and processes such as computer-assisted learning, web-based training, virtual classrooms and digital collaboration (Kashive et al., 2020). is the reason for its popularity results from numerous associated advantages, including (Kashive et al., 2020; Layali & Al-Shlowiy, 2020; Raturi, 2018; Reddy et al., 2020b):

- No limitation of pace and time. Students can access the content and learn at their own speed and time
- Promotion of active learning, as students take the lead role in the learning process
- Promotion of student-centred, self-directed, interactive, flexible learning
- Students are exposed to the use of versatile education tools

Although e-learning comes with a lot of advantages, there are many barriers that the higher education institutes face in the successful facilitation and delivery of e-learning including the network infrastructure, lack of students’ computer competencies, students’ efficacy in using technology for learning, and the potential for miscommunication between students and facilitators (Layali & Al-Shlowiy, 2020; Mousavi et al., 2020; Rafiq et al., 2020). Researchers have also highlighted that since e-learning is closely linked to technology, and because students must use these communication tools for learning, their competency and efficacy in the use of such technology is extremely important (Arshavskiy, 2017; Henderson et al., 2017; Sakarji et al., 2019). Kashive et al. (2020) and Rafiq et al., (2020) note that positive perceptions and attitudes of students toward technology is a strong determinant factor in a successful e-learning system. In the context of this study, student “attitude” can be defined as the “knowledge, feeling and action of an individual towards learning with technology or e-learning. Bhuvaneswari and Dharanipriya (2020) propose that attitude indicates the degree of potential adaptation to technology, and hence a favourable attitude to e-learning would mean that students would be more likely to accept online learning systems. Similarly, in the present study the term “perception” refers to how an individual feel about the use of technology for learning. Previous researchers have proposed that if students perceive that e-learning is useful and helpful to their studies, they will be more likely to accept it (Dospinescu & Dospinescu, 2020; Mahajan, 2020; Sakarji et al., 2019).

Due to the challenges of student acceptance, digital competency and technological acceptance, it could seem at face value that the learning outcomes of students participating solely in online education could be poorer than those engaging in face-to-face learning. However, the jury appears to still be out on whether there are statistically significant differences between the outcomes between these two modes of content delivery. Beinicke and Bipp (2018) reported that across the entire course content, e-learning was just as effective as in-person learning for procedural knowledge and more effective than in-person learning for declarative knowledge. Baxley (2018) argued for a more integrative approach, suggesting that online learning can successfully be used to augment face-to-face teaching methods and improve cohort achievement. However, most studies on the quality of e-learning services have been conducted in developed countries (Dursun et al., 2014; Machado-Da-Silva et al., 2014; Martínez-Argüelles et al., 2013) and it is unclear if the same underlying factors and trends would be similar in developing nations (Pham et al., 2019).

The South Pacific countries are one such group of developing nations, currently undergoing a technological revolution in order to meet the growing digital demands of the populace (Reddy et al., 2016, 2020a, 2020c; Sharma et al., 2019a). Many higher education institutes in the South Pacific have transited to technology enabled learning to make the learning processes more effective and to meet the demands from the students. However, the phenomena of e-learning in the South Pacific is still developing and there are relatively few studies explored student attitudes and perceptions toward the use of technology, with most studies focused on technology and student acceptance (Raturi, 2018; Reddy et al., 2020b; Sharma & Reddy, 2015; Sharma et al., 2020, 2019b).

Both the potential and challenges of e-learning were recently highlighted by the outbreak of the global COVID-19 pandemic in early 2020. As a result, education providers worldwide were forced to turn to e-learning and emergency remote teaching within extremely short timespans. For universities with a traditionally predominant face-to-face delivery mode, this has resulted in many challenges as they scramble to transfer their unit content online (Miltiadous et al., 2020). Nevertheless, with forced campus closures due to local governmental policies, e-learning and to some extent, m-learning (mobile learning) are the only means left for universities to retain their student base and ensure they can continue learning throughout the pandemic. Universities in more regional areas and in developing nations have also been hit hard. In these situations, a considerable number of students may not have access to suitable ICT and/or internet access at their home location, so risk falling behind their peers when attempting the switch to e-learning. Universities that have a long history of online education, such as CQUniversity and Charles Sturt University in Australia, and the University of the South Pacific in the Pacific region, are considerably better off in this regard compared to many of the well-established "sandstone" universities, as the transition from "mostly online" to "fully online" is considerably easier to achieve. Furthermore, their student base is also likely to be better prepared in terms of organising access to suitable technology and internet access, as even the face-to-face units offered by such universities typically have a considerable online component/presence associated with the unit (CQUniversity, 2020; Rapanta et al., 2020).

Since the beginning of the pandemic, many studies have been published documenting university students' readiness towards e-learning and/or learning from home across the globe (Ali, 2020; Chung et al., 2020; Leacock & Warrican, 2020; Naji et al., 2020). However, to the best of our knowledge, there is no published data available on students' e-preparedness from the South Pacific region. Hence, the present study aims to provide insight into the preparedness of first-year students enrolled at the University of the South Pacific towards e-learning in the COVID-19 pandemic context. The University of the South Pacific (USP) occupies a unique status as one of three regional universities in the world, being owned by its 12 member countries: Cook Islands, Fiji Islands, Kiribati, Marshall Islands, Nauru, Niue, Samoa, Solomon Islands, Tokelau, Tonga, Tuvalu and Vanuatu. With a student roll of about 30,000, USP has 14 campuses and 10 study centres spread over an area of 30 million square kilometres, with at least one campus hosted in each member country. The campuses vary in size and student populations, as well as the available support services and facilities, and modality of delivery. The main campus of USP is located in Fiji, which hosts the university's administrative, academic and commercial operations (Sharma & Reddy, 2015; Sharma et al., 2018a, 2019a). The university offers flexible and distance learning programmes delivered through a variety of modes and technologies, although the majority of students are enrolled in face-to-face programmes. While the university is committed to delivering quality education and adequate learning support to students across its member countries, many students have somewhat limited access to computers and broadband internet, making the shift to online learning as a result of the COVID-19 pandemic quite a challenge.

The current study aims to investigate the attitudes and perceptions of USP students towards e-learning, their ICT and information literacy competencies, their self-efficacy and their confidence in using technology for learning. It is envisaged that our findings will be useful not only for education providers in the oceanic region but also for regional governments and other education stakeholders in developing nations more globally. Specifically, we aim to address the following research questions:

1. What is the current status of ICT (technological) literacy for the South Pacific students?
2. What is the current status of information literacy for the South Pacific students?
3. How frequently do students participate in various ICT activities?
4. How confident are students in using ICT products?
5. What are current student attitudes and perceptions toward the use of computers?

## Methods

This study uses data collected as part of a PhD study on eLearning and digital literacy by one of the authors (PR). Research and ethics approval was provided by the USP research office. The survey instrument used in this study was a unipolar Likert scale 1–5 questionnaire administered to the students using Google Forms. A copy of the survey instrument is provided in the Additional file 1. The questions were designed to gain an overview of the e-learning resources available to the students (e.g. type of electronic devices available; type of internet connection), the preparedness of students toward e-learning (e.g. length of time they have been using computers for, amount of time spent on the internet per day, level of ICT troubleshooting skills) and student perceptions or thoughts toward the use of ICT and e-learning. We refer to these aspects as "ICT resources", "ICT literacy" and "ICT perceptions" throughout this paper.

Our study population was first-year undergraduate students enrolled at the University of the South Pacific. Students were surveyed in February 2020 prior to the commencement of their first year of university study. All commencing students at the University of the South Pacific were invited to complete the survey and were given a window of approximately three weeks in which to do so. A total of 313 students opted to provide informed consent and complete the survey. At the closing time of the survey (24 February), there were approximately 83,000 confirmed cases of COVID-19 globally, with less than 5000 cases outside of China (World Health Organization, 2020). There were no known cases of COVID-19 in any Pacific Island country or territory at this point, with the first case reported in French Polynesia around 17 days later (Craig et al., 2020). Hence we consider it unlikely that the COVID-19 pandemic impacted on the student responses in this survey, thus providing an accurate insight into the e-preparedness of first-year students who were predominantly expecting to study face-to-face in the upcoming semester.

In general, the response of USP in moving its teaching and learning online was not a major issue following the outbreak of the COVID-19 pandemic, as many courses offered by the university were already facilitated online for distance students. However, emergency remote teaching was a challenge for the university due to the persisting issues including network infrastructure and student access to suitable e-devices and the internet. The unit facilitators encountered many challenges during emergency remote teaching, including the design of assessments and exams to minimise the potential for online teaching, monitoring students' progress, and adjusting assessment due dates to accommodate students whose study had been disrupted as a result of difficulty accessing the internet or other external factors.

The questions that involved a quantizable response (e.g. "how often do you perform the following activities", "how important are the following", "rate yourself on these skills", or "how much do you agree with these statements") were numerically coded in a similar fashion to a Likert scale, ranging from 1 (never/not important at all/no capability/strongly disagree) to 5 (frequently/very important/excellent/strongly agree). Statistical analyses were performed in R Studio, running R 4.0.2 (R Core Team, 2020). Students who did not respond to demographic data such as their age bracket, country or program were excluded from subsequent data analysis, leaving a total of 295 valid responses. Students who did not respond to a specific question were excluded from analyses pertaining to that question only.

## Results and discussion

### Demographics

**Fig. 1:**
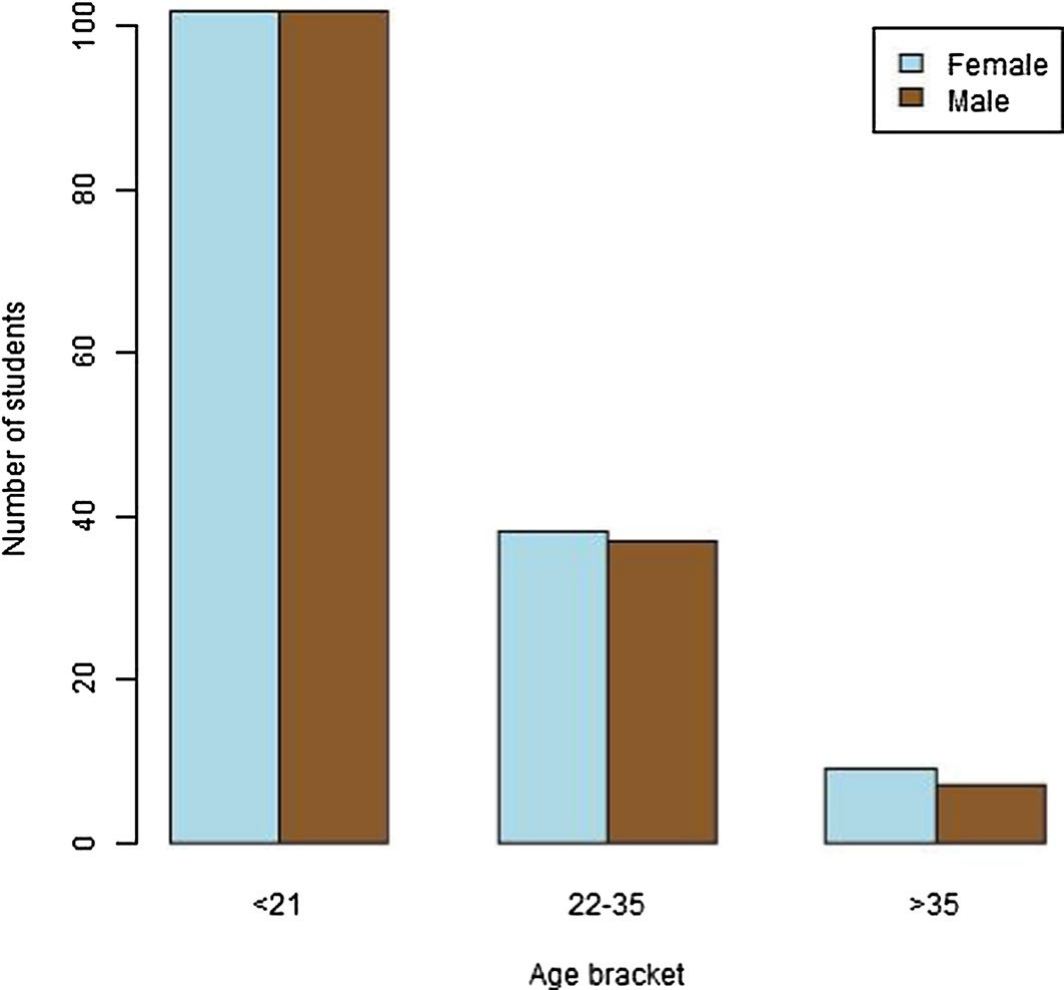
Barplot showing the numbers of male and female students in each age bracket

**Fig. 2:**
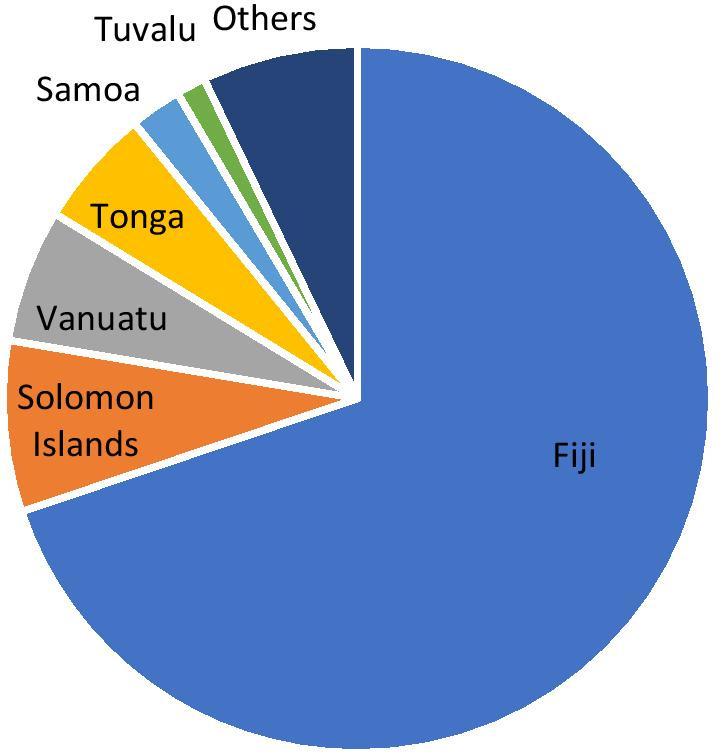
Breakdown of the survey respondents by their country of residence

The number of male and female survey respondents were virtually equal, at 49.5% and 50.5% of the total respondents, respectively (Fig. 1). The majority of respondents were studying a Science program (60%), followed by Arts (30.8%) and Education (9.2%). Approximately 69.2% of the freshmen students were 21 years of age or under, with 25.4% between 22–35 years of age and the over 35 years age bracket making up the remaining 5.4% of students (Fig. 1). The number of respondents from the different Pacific Island countries is illustrated in Fig. 2. The majority of students (69.8%) were from Fiji, with the next highest numbers of students from the Solomon Islands (7.8%), and Vanuatu (6.1%). Most of these students would normally study face-to-face, with a minority studying online/via distance.

### ICT resources

**Fig. 3:**
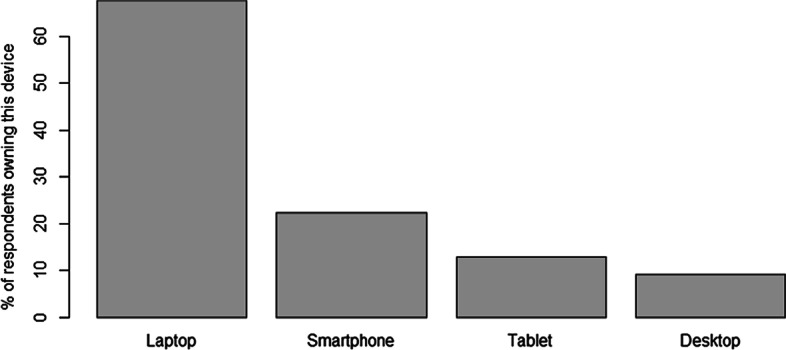
ICT device ownership among the surveyed students. Note that the total percentages do not sum to 100% as some respondents owned more than one type of device

Of the 287 students who provided a valid response as to which ICT devices they own, 88.2% of all students reported owning at least one ICT device. The likelihood of a student owning at least one ICT device was not influenced significantly by their gender, age bracket, nationality or program of study (Chi-square test or Fisher's exact test; P > 0.05 for all). The majority of students (67.6%) owned a laptop, with approximately 9% of students owning a desktop computer and 13% owning a tablet or iPad (Fig. 3). Only 22.3% of students reported owning a smartphone in the present study. However, we consider that this low figure is due to most students not interpreting the question ("do you own an ICT device of your own?") as referring to smartphones. Indeed, it has previously been reported that the ownership of mobile devices in the PICs in 2014 was 93% (Reddy et al. 2016), which seems more reasonable.

The most common type of internet connection that students reported access to was mobile internet service (57.3% of students), followed by Wi-Fi connection (29.5%). Only 5.9% of students reported access to a Broadband internet service at home, while 7.3% reported access to a Broadband internet service at their university campus. Prior studies conducted in the South Pacific reveal that more people now have access to the internet, with subscriptions increasing every year due to the falling price of internet services and improvements in the network infrastructure (Reddy et al., 2017, 2020a, 2020b; Sharma & Reddy, 2015; Sharma et al., 2020). However, persisting connectivity issues impact on how students access the internet or which internet connection they use (Reddy et al. 2017, 2020b). This has resulted in a growing trend toward the use of mobile devices in the South Pacific, as a result of the falling prices and more affordable data plans for mobile devices (Reddy et al., 2016). Consequently, surveys conducted among South Pacific students indicated that 90% are in favour of using their mobile phone for learning purposes (Reddy et al., 2017), in a trend known as mobile learning (m-learning). This has significant implications for education providers and stakeholders with interest in improving education access equity. Typically, mobile internet access will be slower and more variable compared to Wi-Fi or Broadband access. Furthermore, the data costs (per gigabyte) can be much higher than satellite or cable internet access. On the whole, students who can only access the internet through a mobile phone service are likely to have more restricted access to the internet in general. This can make it harder for them to access online content material, particularly when associated with data-heavy applications such as streaming lecture videos or accessing live tutorial videoconferences.

More recently, government initiatives of providing free Wi-Fi in countries such as Fiji, Vanuatu, Cook Islands, Niue and the Solomon Islands – particularly at higher education centres – have shown an increase in Wi-Fi use. Furthermore, we believe that an increase in internet usage and e-device ownership will continue to increase in the education sector, particularly due to the mandatory shift from the traditional method of face-to-face learning to online learning during and post COVID-19 pandemic. From our personal observations in South Pacific countries, the onset of the pandemic has resulted in internet providers lowering data plan prices, in some cases in collaboration with South Pacific educational institutes to ensure all students have adequate internet access to continue their learning journey.

### Student ICT literacy

**Fig. 4:**
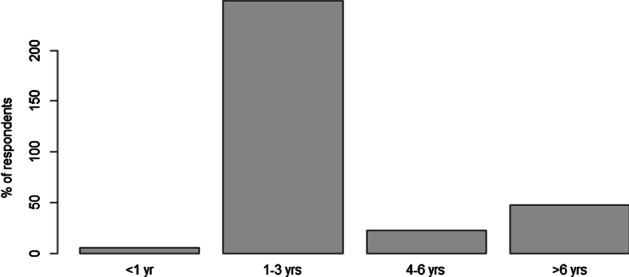
Number of years of experience using ICT devices among the surveyed students

Approximately 47.1% of the students reported having used computers and/or the internet for a time period of longer than 6 years prior to the survey (Fig. 4). Only 5.5% of students had used ICT devices for less than one year. However, there was a significant interaction between the length of time students had used ICT and their likelihood of owning at least one ICT device (Fisher's Exact test; P < 0.05). Students who had used ICT for 1–3 years were much less likely to own an ICT device than expected by chance, while students who had used ICT for over 3 years were more likely to own an ICT device.

The majority of students (60.7%) reported spending between 1–4 h per day on the internet for university or school-related purposes, with around a fifth spending 5–8 h per day on these activities (Table 1). Over 6% of students used the internet for over 9 h each day for university purposes. Slightly more students than this (9.8%) used the internet for over 9 h each day for edutainment, which can be loosely interpreted as media intended to be both educational and enjoyable. As defined in the survey provided to students, this includes purposes such as games, music, videos and social networks. Students' distribution across the other three-time brackets for edutainment followed an approximately normal distribution, with almost half (46%) spending 1–4 h on it per day.

**Table 1:** Percentage of students using the internet for the specified time brackets for each purpose (school or edutainment) per day

Time spent (university)	Time spent (edutainment)				
	< 1 h	1–4 h	5–8 h	> 9 h	Sum
< 1 h	3.1%	4.5%	1.0%	1.7%	10.5%
1–4 h	12.2%	27.4%	17.0%	5.2%	60.7%
5–8 h	5.6%	11.1%	3.5%	1.0%	20.7%
> 9 h	0.7%	3.1%	1.0%	1.7%	6.4%
Sum	21.0%	45.8%	22.4%	9.8%	

Further breakdown of time spent on the internet revealed that students reported their most frequent activity as email, followed by accessing online educational resources and communication (through non-email means such as Skype, Viber, Facebook or online forums) and social networking (Table 2). The majority of students reported accessing their online educational resources "very often" and reading free course content "sometimes".

**Table 2:** Break-down of the student cohort by how frequently they participate in the listed ICT activities

Activity (no. of respondents)	Never	Rarely	Sometimes	Very often	Frequently
*Email* (n = 287)	0%	3.5%	21.2%	28.1%	47.2%
*Downloading music & video* (n = 280)	3.8%	18.9%	36.0%	18.5%	22.7%
*Communication (non-email)* (n = 288)	1.4%	6.3%	20.6%	32.2%	39.5%
*Social networking* (n = 288)	2.1%	6.3%	26.9%	30.4%	34.3%
*Reading wikis* (n = 286)	12.1%	26.4%	35.7%	18.2%	7.5%
*eBooks* (n = 283)	16.7%	27.3%	32.3%	14.2%	9.6%
*Reading free course content* (n = 286)	4.5%	12.2%	35.9%	32.4%	15.0%
*Learning management system* (n = 282)	9.5%	15.5%	35.6%	24.7%	14.5%
*Digital library* (n = 283)	12.5%	21.8%	33.9%	21.4%	10.4%
*Playing games* (n = 286)	17.0%	21.2%	27.9%	17.0%	17.0%
*Online educational resources* (n = 280)	0.7%	4.2%	19.8%	44.4%	30.9%
*Attending online mentoring* (n = 286)	9.8%	18.9%	26.9%	30.4%	14.0%

A common definition of ICT literacy is "an individual's ability to use computers to investigate, create, and communicate in order to participate effectively at home, at school, in the workplace, and society" (Fraillon et al., 2013). Hence, it is informative to assess the length of time spent and activities performed by students using ICT and their efficacy at performing various common ICT-related tasks. In general, the students in this cohort reported strong skills in computers' technical use (Table 3). Their average information literacy was a little lower compared to their technical capabilities. Still, it ranged between "good" and "excellent" for the majority of students. In the South Pacific the concept of information literacy is growing. For example, the current cohort of university students surveyed for this study have to complete a compulsory information literacy unit in their first year of their study (Reddy et al., 2020d). Therefore, the current student cohort's information literacy competencies should also improve significantly throughout their study when compared to their other literacies.

**Table 3:** Self-reported ratings of students for the technical use of computers and their information literacy, with a particular focus on the internet

Activity (no. of respondents)	Fair	Good	Excellent
Technical use of computers			
Turning on a computer (n = 288)	1.7%	11.5%	86.8%
Customising desktop environment (n = 276)	10.5%	25.7%	63.8%
Opening a file (n = 288)	2.8%	14.6%	82.6%
Copying and deleting a file (n = 288)	1.7%	11.5%	86.8%
Organising and managing files (n = 281)	6.8%	23.5%	69.8%
Connecting to Internet (n = 288)	3.5%	20.8%	75.7%
Installing a program (n = 254)	16.5%	28.7%	54.7%
Printing a document (n = 266)	10.9%	25.2%	63.9%
Placing an image on a document (n = 270)	10.7%	20.4%	68.9%
Using MS Word/Excel/PowerPoint (n = 268)	12.7%	28.4%	59.0%
Information literacy			
Downloading files (n = 274)	5.5%	19.7%	74.8%
Saving image from web (n = 279)	6.8%	16.8%	76.3%
Emailing (n = 282)	7.4%	21.3%	71.3%
Creating web pages (n = 207)	37.2%	36.2%	26.6%
Sending attachments (n = 263)	10.6%	26.2%	63.1%
Able to use search engines (n = 265)	8.7%	20.0%	71.3%
Using keywords to find information (n = 268)	11.2%	29.5%	59.3%
Using given URL to look for information (n = 273)	15.0%	25.6%	59.3%
Using bookmark (n = 252)	23.4%	30.2%	46.4%
Using advanced search (n = 248)	22.6%	33.5%	44.0%
Navigating through web pages (n = 264)	14.8%	32.2%	53.0%
Internet browsing (n = 282)	4.6%	25.2%	70.2%
Presentation tools (n = 253)	22.9%	37.5%	39.5%
Participating in social networks (n = 269)	20.4%	30.9%	48.7%
Using the Internet safely (n = 270)	16.3%	30.7%	53.0%
Using information from the Internet without plagiarising (n = 267)	23.2%	33.3%	43.4%
Judging the reliability of information on the Internet (n = 258)	21.3%	40.7%	38.0%

### Student attitudes towards ICT

The majority of students (55.6%) reported either "loving" or "liking" new technology and that they were usually the first to try new technology among their acquaintances. Only 19% of students reported that they "were not used to new technology" or were "one of the last people to try new technology", suggesting an overall positive attitude and interest towards ICT among the student cohort.

The most important uses of e-learning, as reported by the student cohort, were for video/audio tutorials, email groups and open education resources (Table 4). Only a few students reported the use of education games, blogs, social networking sites or wikis/podcasts for education purposes. Most students were moderately to strongly confident in their abilities to use technology, although not necessarily with troubleshooting computer problems (Table 5).

**Table 4:** Importance of various online resources related to e-learning, as reported by the student cohort

Importance (no. of respondents)	Not important at all	Not important	Neutral	Important	Very important
Email groups (n = 288)	0.3%	0%	10.8%	34.4%	54.5%
Video and audio tutorial (n = 288)	0%	0%	4.9%	24.0%	71.2%
Blogs (n = 258)	1.9%	0%	46.5%	34.9%	16.7%
Social Networking sites (n = 264)	1.5%	0%	37.1%	40.9%	20.5%
Wikis and podcast (n = 260)	3.5%	0%	42.3%	36.9%	17.3%
eBooks (n = 267)	4.5%	0%	34.8%	37.8%	22.8%
Open Educational Resources (n = 287)	0.3%	0%	8.4%	37.6%	53.7%
Learning Management System (n = 278)	0.7%	0%	17.3%	37.4%	44.6%
Digital Library (n = 270)	1.1%	0%	19.3%	40.7%	38.9%
Education games (n = 263)	3.8%	0%	34.6%	39.5%	22.1%
Attending online mentoring (n = 284)	0.4%	0%	20.1%	39.4%	40.1%

**Table 5:** Self-reported confidence of students in the use of ICT and e-learning

Statement (no. of respondents)	No capability	Low capability	Fair	Good	Excellent
I feel confident in using computers (n = 288)	0%	1.0%	5.9%	30.6%	62.5%
I feel confident in using the computer organize tools (n = 282)	0%	1.4%	13.5%	40.4%	44.7%
I feel confident in organizing and managing files (n = 284)	0%	1.4%	12.7%	33.5%	52.5%
I feel confident troubleshooting computer problems (n = 281)	0.4%	7.5%	30.6%	32.7%	28.8%
I can complete the required task using the learning tools if I have the manuals for reference (n = 284)	0%	2.5%	16.5%	33.1%	47.9%
I can complete the required task using the learning tools if I have the built in facility for assistance (n = 283)	0.4%	2.1%	16.3%	38.2%	43.1%
I can complete the required task using the learning tools if someone showed me how to do it first (n = 283)	0%	0.4%	7.4%	32.2%	60.1%
I enjoy using ICT for my studies (n = 280)	0%	0.7%	8.2%	27.9%	63.2%
I believe that e-learning gives me the opportunity to acquire knowledge (n = 282)	0%	0.4%	7.1%	26.6%	66.0%
I believe the e-learning enhances my learning experience (n = 281)	0%	0.7%	6.4%	26.3%	66.5%
I believe that e-learning increases the quality of learning because it integrates all forms of media (n = 282)	0.4%	0.7%	7.8%	31.9%	59.2%
I feel satisfied with my learning content by adopting to e-learning technology (n = 283)	0.4%	0.7%	12.0%	36.0%	50.9%
I would be interested in studying courses that use e-learning (n = 280)	0.7%	1.1%	15.4%	28.6%	54.3%

Virtually all students reported a positive attitude toward the use of technology in the learning process, particularly for keeping connected to their course material and accessing a wide range of educational resources (Table 6).

**Table 6:** Student attitudes and perceptions toward the use of technology in the learning process

Statement (no. of respondents)	Strongly disagree	Disagree	Neutral	Agree	Strongly agree
Technology gives me access to a wide range of learning materials (n = 285)	0%	0%	2.1%	15.8%	82.1%
Technology keeps me connected to the courses I am enrolled in (n = 282)	0%	1.4%	13.5%	40.4%	44.7%
Technology connects me with my peers and facilitator (n = 284)	0%	1.4%	12.7%	33.5%	52.5%
Technology will help me to complete my work faster (n = 281)	0.4%	7.5%	30.6%	32.7%	28.8%
Technology will help me to produce quality work (n = 284)	0%	2.5%	16.5%	33.1%	47.9%
Technology makes learning creative (n = 283)	0.4%	2.1%	16.3%	38.2%	43.1%
Technology will enable me to take control of my learning (n = 283)	0%	0.4%	7.4%	32.2%	60.1%

### General discussion

The current study shows that of the 88% of participants who own at least one ICT device, all have access to the internet in some form, whether it be through mobile data, Wi-Fi or broadband, either on campus or at home. In comparison with prior such studies, the study noted an increase in device ownership and Internet accessibility for the South Pacific countries. Given that all participants of this study were students from USP, they would also have access to university computer labs across all campuses which can be utilised for learning purposes, in addition to their personal e-devices. Furthermore, the university also provides free Wi-Fi access at all campuses so that students who do not have personal access to the internet can use the services provided at the campuses. Overall, these are quite positive trends toward the uptake of digital technologies across the South Pacific region and the current and future potential for e-learning. This concurs with previous studies reporting that technology-enabled learning has been well received by most students (Raturi, 2018; Reddy et al., 2019; Sharma et al., 2020, 2019b). Although there have been drawbacks, as discussed in the introductory sections, initiatives to improve the facilitation of technology-enabled learning from the higher education stakeholders in the South Pacific have been put in place by government agencies and researchers. These include the provision of ICT devices and training to students in need.

In terms of the technological competency of the students, the overall response was noted to be above average, indicative of widespread technological acceptance. The results for the usage of digital technology indicated that the participants mostly used their technology for accessing and sending emails, followed by accessing online educational resources and social networking websites. The high competency of students in using emails is prominent as a result of all USP students being issued with a university email address. Similarly, USP facilitates at least some portion of all courses through an online learning management system (Moodle), thus accessing educational resources through this platform is expected to be a regular part of their study experiences. In contrast, students’ usage of digital library resources and free online course content was considerably lower, predominantly ranging from “rarely” to “sometimes” in the former instance. Hence, we believe that as commencing first-year students, they may have lacked knowledge about these aforementioned resources. A study conducted by Reddy et al. (2020c) similarly revealed that students in the South Pacific lack digital literacy knowledge, suggesting that more outreach efforts and awareness programs are needed to expose the students to the latest digital literacy trends. In other words, students from the South Pacific appear to generally have sufficient technological skills, but may require further support with their digital literacy (finding academically sound information, judging its reliability, etc.). Various initiatives have been put in place in the South Pacific to impart and solidify students’ computer skills, including:

i. A framework for Pacific Regional Framework 2018–2030 – Moving Towards Education 2030, which includes increasing students’ digital literacy (Pacific Islands Forum Secretariat, 2018)
ii. The National Framework of Digital Literacy in Fiji, which includes programmes for students from Year 1 – Year 12
iii. The SchoolNet Project in Samoa to incorporate e-learning and increase the use of computers at secondary and primary schools, run by the Pacific Region Infrastructure Facility
iv. Pilot trial of one laptop per child (OLPC) initiative in Fiji, Samoa, Solomon Island, Tonga and Vanuatu, run by the Pacific Region Infrastructure Facility
v. Provision of ICT training by the USP to around 860 primary school students and 680 parents in the Western division of Fiji (Yusuf, 2009)

Overall, the attitude of the majority of students towards online learning was positive. The students perceived that online learning connected them more with their peers and facilitators, gave them access to wider range of learning materials, made the learning process more creative and enabled self-paced learning. This agrees with a recent study conducted in the South Pacific by Reddy et al., (2020a), who found that the aforementioned benefits of online learning resulted in the positive attitudes toward technology, ensuring that students were open to continued use of e-learning.

As the present study was conducted just prior to the arrival of COVID-19 in the South Pacific and before all higher education institutes had to move their delivery mode online (USP, 2020a), it provides an insight into the “typical” level of e-preparedness among students from the Pacific Island Countries. The majority of students in this study showed high levels of e-preparedness, indicating that they would be expected to be somewhat ready for the transition from face-to-face to online learning. This agrees with anecdotal observations during the pandemic, where it was reported that the semester and exams were able to progress via an online format with minimal disruptions and participation rates of up to 80% (USP, 2020b). In contrast, Chung et al. (2020) reported that over 50% of Malaysian students did not wish to continue with e-learning following the COVID-19 pandemic. The key challenges identified by these students were internet connectivity, a lack of continuity in the online delivery methods used by different lecturers, and difficulty understanding online content provided by lecturers (Chung et al., 2020). This highlights the digital competency of university educators and staff as another important facet of e-learning (Ali, 2020). Indeed, high levels technological and digital literacy among educators was identified by several authors to be a key factor in the successful transition to online learning (Chung et al., 2020; Naji et al., 2020; Rapanta et al., 2020).

Despite these promising results found amongst students from the South Pacific, ongoing work is necessary to identify and assist students experiencing difficulty in accessing and using digital technology to assist their learning process. This highlights the importance for universities to maintain awareness of the e-capabilities of their student population, perhaps through the use of surveys such as the one presented in this study, or the Student Experience of Learning and Teaching (SELT) survey. This will assist these institutions in delivering a robust system of education that can adapt to future challenges.

## Conclusion

The increasing use of online modes of content delivery has the benefit of increasing convenience for students, by allowing them to choose where and when to learn, but also has the potential to improve equitable access to education by providing students in geographically remote locations with the option to pursue tertiary education without having to relocate to a more urban locality. However, a number of challenges to this approach remains, including ensuring equitable access to suitable technology and internet connections, maintaining high quality in online learning resources and ensuring the learning outcomes of online students are comparable to those achieved through face-to-face classes.

Following the recent outbreak of COVID-19, education providers worldwide have been forced to turn to e-learning to retain their student base and allow them to continue learning through the pandemic. However, in geographically remote, developing nations, many students may not have access to suitable technology or internet at their home location but are rather dependent upon face-to-face classes at their local university campus to allow them to participate in higher education.

This study found that among the commencing 2020 cohort of students at the University of the South Pacific, 88% possessed at least one ICT device and access to the internet. Similarly, most students had adequate to strong ICT skills and a positive attitude toward e-learning. These attitudes among the student cohort, in conjunction with the previous experience of The University of the South Pacific in distance education, are likely to have contributed to the relatively successful transition from face-to-face to online learning as a result of the COVID-19 pandemic. Educational institutes should maintain ongoing awareness of the e-capabilities of the student cohorts in order to be best prepared for future challenges to the educational sector.

## Supplementary Information

**Additional file 1.** Survey Instrument.

## Data Availability

The dataset is available from the authors upon request.
