# Supplementary material for: Attitudes and awareness of regional Pacific Island students towards e-learning
Source: Int J Educ Technol High Educ. 2021 Mar 11;18(1):13. doi: 10.1186/s41239-021-00248-z (PMC7946445; doi:10.1186/s41239-021-00248-z)
Supplement: Supplementary file 1 — Additional file 1. Survey Instrument. [file 41239_2021_248_MOESM1_ESM.pdf]

Faculty of Science, Technology and Environment

Transforming Higher Education in the South Pacific using

E-Learning Technologies and Digital Literacy

(Note: By filling this questionnaire you agree to participate in this research project. Personal identification details are not asked in this survey therefore you remain anonymous. All responses will be confidential, compiled and analysed as a group.)Please circle or tick where necessary.

School/ Institute: _____________________________ | Form/Year: _______ | Gender: ◌ Male ◌Female
Age: ______ | School Location: ◌ Urban ◌Rural | Country: ______________
Programme/Subject Area: ___________________________________

1. How long have you been using computers/ and internet?
   ☐ Less than 1 year ☐ 1-3 yrs ☐ 4-6 yrs ☐ >6yrs

2. Do you own an ICT device of your own? If yes, which one?
   ____

3. Which statement best describes you?
   a. I love new technology and I am usually the first one to try them.
   b. I like new technology and I like to try to before the people I know.
   c. I use new technology when I know people whom I know use them.
   d. I am one of the last people to try new technology.
   e. I am not used to new technology, I use if I have to.

4. How much time do you spend doing the following activities per day using internet?

	School Activities	Edutainment (Games, Music, Videos, Social Networks)
< 1 hour		
1-4 hours		
5-8 hours		
>9 hours		

5. What type of Internet connection do you have access to?
☐Broadband home ☐Broadband school/university
☐Wi-fi connection ☐Mobile Internet

6. Which of the following activities do you perform using internet and how often?

	Frequently	Very Often	Sometimes	Rarely	Never
Email					
Downloading music and video					
Communication (Skype, Viber, Facebook, Online forums)					
Social Networking					
Reading wikis					
ebooks					
Reading free course content					
Learning Management System					
Digital Library					
Playing games					
Accessing Online Educational Resources					
Attending online mentoring					

7. How important are the following?

	Very Important	Important	Neutral	Not very important	Not important at all
Email groups					
Video and audio tutorial					
blogs					
Social Networking sites					
Wikis and podcast					
ebooks					
Open Educational Resources					
Learning Management System					
Digital Library					
educational games					
Attending online mentoring					

8. What are some of the challenges affecting the use of ICTs for learning?

____
____
____

9. What are some positive and negative implications of learning with ICT.

Positive:____
____
____

Negative:
____
____
____

10. Do you think learning with ICT is a good idea? State with reasons.

____
____
____
____

11. How will you rate yourself in the following?

**Variables**	**Excellent**	**Good**	**Fair**	**Low capability**	**No Capability**
**Use of Computers (Technical)**					
Turning on a computer					
Customising desktop environment					
Opening a file					
Copying and Deleting a file					
Organizing and managing files					
Connecting to Internet					
Installing a program					
Printing a document					
Placing an image on a document					
Using Microsoft word/ excel and powerpoint					
**Information Literacy (Finding and Evaluating Information)**					
Downloading files					
Saving image from web					
Emailing					
Creating web pages					
Sending attachments					

Able to use search engines					
Using keywords to find information					
Using given URL to look for information					
Using bookmark					
Using advance search					
Navigating through web pages					
Navigating through files					
Internet Browsing					
Presentation tools					
Participating in social networks					
Using the Internet safely					
Using information from the Internet without plagarising					
Judging the reliability of the information on the Internet					

12. Rate using the scale: 5- Strongly Agree, 4- Agree, 3- Neutral, 2- Disagree, 1- strongly disagree

i. I feel confident in using computers __________
ii. I feel confident using the computer organize tools _________
iii. I feel confident in organizing and managing files __________
iv. I feel confident troubleshooting computer problems __________
v. I can complete the required task using the learning tools if I have the manuals for reference ___________
vi. I can complete the required task using the learning tools if I have the built in facility for assistance _____________
vii. I can complete the required task using the learning tools if someone showed showed me how to do it first ______________
ii. I enjoy using ICT for my studies _______________
iii. I believe that e-learning gives me the opportunity to acquire new knowledge __________
iv. I believe that e-learning enhances my learning experience __________
v. I believe that E-learning increases the quality of learning because it integrates all forms of media ____________
vi. I feel satisfied with my learning content by adopting to E-Learning Technology ____
vii. I would be interested in studying courses that use e-learning _________

13. Rate using the scale: 5- Strongly Agree, 4- Agree, 3- Neutral, 2- Disagree, 1- strongly disagree

a. Technology gives me access to wide range of learning materials. ________
b. Technology keeps me connected to the courses I am enrolled in. _________
c. Technology connects me with my peers and facilitator. __________
d. Technology will help me to complete my work faster. ___________
e. Technology will help me to produce quality work. _____________
f. Technology makes learning creative. _____________
g. Technology will enable me to take control of my learning. ______________

Thank you very much for taking part in this survey
